# Dog-Owner Attachment Is Associated With Oxytocin Receptor Gene Polymorphisms in Both Parties. A Comparative Study on Austrian and Hungarian Border Collies

**DOI:** 10.3389/fpsyg.2018.00435

**Published:** 2018-04-05

**Authors:** Krisztina Kovács, Zsófia Virányi, Anna Kis, Borbála Turcsán, Ágnes Hudecz, Maria T. Marmota, Dóra Koller, Zsolt Rónai, Márta Gácsi, József Topál

**Affiliations:** ^1^Institute of Cognitive Neuroscience and Psychology, Research Centre for Natural Sciences, Hungarian Academy of Sciences, Budapest, Hungary; ^2^Comparative Cognition & Wolf Science Center, Messerli Research Institute, University of Veterinary Medicine, Vienna, Medical University of Vienna, University of Vienna, Vienna, Austria; ^3^Department of Ethology, Eötvös University, Budapest, Hungary; ^4^Department of Medical Chemistry, Molecular Biology and Pathobiochemistry, Semmelweis University, Budapest, Hungary; ^5^MTA-ELTE Comparative Ethology Research Group, Budapest, Hungary

**Keywords:** oxytocin, dog *(Canis familiaris)*, owner, attachment, relationship, personality

## Abstract

Variations in human infants' attachment behavior are associated with single nucleotide polymorphisms (SNPs) in the oxytocin receptor (OXTR) gene, suggesting a genetic component to infant-mother attachment. However, due to the genetic relatedness of infants and their mothers, it is difficult to separate the genetic effects of infants' OXTR genotype from the environmental effects of mothers' genotype possibly affecting their parental behavior. The apparent functional analogy between child-parent and dog-owner relationship, however, offers a way to disentangle the effects of these factors because pet dogs are not genetically related to their caregivers. In the present study we investigated whether single nucleotide polymorphisms of pet dogs' OXTR gene (−213AG,−94TC,−74CG) and their owners' OXTR gene (rs53576, rs1042778, rs2254298) are associated with components of dog-owner attachment. In order to investigate whether social-environmental effects modulate the potential genetic influence on attachment, dogs and their owners from two different countries (Austria and Hungary, *N* = 135 in total) were tested in a modified version of the Ainsworth Strange Situation Test (SST) and questionnaires were also used to collect information about owner personality and attachment style. We coded variables related to three components of attachment behavior in dogs: their sensitivity to the separation from and interaction with the owner (Attachment), stress caused by the unfamiliar environment (Anxiety), and their responsiveness to the stranger (Acceptance). We found that (1) dogs' behavior was significantly associated with polymorphisms in both dogs' and owners' OXTR gene, (2) SNPs in dogs' and owners' OXTR gene interactively influenced dog-human relationship, (3) dogs' attachment behavior was affected by the country of origin, and (4) it was related to their owners' personality as well as attachment style. Thus, the present study provides evidence, for the first time, that both genetic variation in the OXTR gene and various aspects of pet dogs' environmental background are associated with their attachment to their human caregivers.

## Introduction

Attachment is an organizational construct that serves to organize the development of emotional bond between human infants and their caregivers (Bowlby, 1958). In early infancy its function is to obtain protection and care from another person by adapting one's behavior to characteristics of the key attachment figure (Bowlby, 1969). This early development results in different attachment styles that can be assessed in terms of two dimensions of security/insecurity: attachment-related anxiety and attachment-related avoidance (e.g., Ainsworth et al., 1978; Brennan et al., 1998; Fraley and Spieker, 2003). Attachment styles have well-documented cognitive, physiological, and neurological correlates (e.g., Diamond, 2001; Gillath et al., 2005), and behavioral and psychological consequences that last into adulthood, including self-regulation of stress and emotions, influence on relationship quality with romantic partners, sexual motivation, and reactions to relationship breakups or losses (see Shaver and Clark, 1994; Mikulincer et al., 2003; for reviews).

Most studies have focused on the environmental effects shaping attachment (such as parental behavior, Fearon et al., 2014). more recently however, candidate gene studies have reported associations between attachment styles of human infants and polymorphisms in their dopamine D4 receptor, serotonin transporter, and oxytocin receptor (OXTR) genes (Lakatos et al., 2000; Barry et al., 2008; Chen et al., 2011; Spangler, 2011), suggesting that genetic polymorphisms may moderate the links between parental behavior and other environmental effects and infant attachment. Therefore, it has become obvious that attachment styles are shaped by a combination of genetic factors and social experiences (Fonagy, 2001). This is well-demonstrated by the fact that carrying a specific genetic polymorphism can be associated with developing a particular attachment style in one kind of social environment but not in another (Gillath et al., 2008). For instance, Chen et al. (2011) found that the A allele, as compared to the G allele, of *OXTR* rs2254298 was more likely associated with secure attachment in a non-Caucasian sample but not in a Caucasian sample. Such results in human infants allow for limited conclusions however, because of the genetic relatedness of the infants and their parents. Allelic variations associated with different attachment styles of infants have been shown to affect also various characteristics of the parents, allowing for an alternative, indirect, link between genotype and infant attachment.

In human subjects variations of oxytocin-related genes have been found in association with various personality traits, their components and neurological correlates, such as agreeableness extraversion, social loneliness, anxiety, and amygdala volume (Lucht et al., 2009; Saphire-Bernstein et al., 2011; Haram et al., 2014; Wang et al., 2014). Nonhuman examples also can be found, namely cats with the A allele in the SNP G738A show significantly higher “Roughness” personality scores than cats without the A allele (Arahori et al., 2016). Parental personality affects parenting style (Council et al., 1988; Kendler et al., 1997; Metsäpelto and Pulkkinen, 2003), thereby personality traits have an impact on the quality of parent-child relationships, and on the children's attachment style (Kochanska et al., 2004). However, as infants and their parents likely carry similar alleles in the polymorphic regions of their OXTR, in humans it is difficult to dissect whether and how infant genotypes, parent genotypes and other characteristics of parents (e.g., their personality or their own attachment style) affect infant attachment. The domestic dog, however, provides a unique opportunity to investigate this question.

Dogs have been part of human societies for longer than any other domestic species (Clutton-Brock, 1999). Their ability to form attachment with humans is one of the most widely recognized consequences of domestication (Topál and Gácsi, 2012). Topál et al. (1998) were the first to reveal that dogs develop attachment to their owners analogous to the infant-mother attachment in humans (for a replication see Prato-Previde et al., 2003).

Similarly to human infants, the most widely used paradigm to investigate dogs' attachment behavior toward their owners is the Strange Situation Test (SST; Topál et al., 1998). The test consists of seven episodes, each lasting 2–3 min, when the dog is either with the primary caregiver (owner), with a stranger, or alone in an unfamiliar place. The essential element of the test is that separation from the human caregiver in an unfamiliar environment evokes moderate stress, which manifests in proximity seeking while the reunion with the caregiver evokes contact-seeking behaviors. Multivariate analysis of Topál et al. data (1998) (factor and cluster analyses) separated three key aspects of dogs' behavioral structure. These major factors revealed that dogs' behavior during the test was affected by: (i) their sensitivity to the separation from the owner (Attachment), (ii) the degree of stress the unfamiliar environment evoked from them (Anxiety), and (iii) their responsiveness to the stranger (Acceptance). Their study demonstrated that adult dogs show specific patterns of attachment behavior toward their owners, and dogs' individual behavior patterns can be explained by the different combinations of these determining factors. Many studies have reported that dogs show a great variability in their behavior in the SST (Topál et al., 1998; Gácsi et al., 2001; Naderi et al., 2002; Prato-Previde et al., 2003; Mariti et al., 2014; Scandurra et al., 2016) suggesting that adult dogs are particularly suitable subjects for studying the phenomenon of animal-to-human attachment.

Pet dogs also offer a good model for investigating to what extent attachment patterns are shaped by the independent genetics of the dogs and their owners and by environmental factors, such as the owners' personality, attachment style, or the country they live in. Drawing a parallel between infant-mother and dog-owner attachment has recently gained further support from the finding that ownership styles seem to be composed of components similar to those of human parenting behavior, suggesting that owners largely use their parenting repertoire when interacting with their dogs (Cimarelli et al., 2017). Importantly, however, only little (and indirect) data is available on how different genetic and environmental factors influence dogs' attachment to human.

Similarly to the finding that polymorphism in the OXTR gene is related to security/insecurity of mother-infant attachment in humans (Chen et al., 2011), it has been suggested that oxytocin plays an important role in the relationships between dogs and their owners, with higher oxytocin levels being associated with a more positive relationship from perspective of the owner (Thielke and Udell, 2015). Thielke and Udell even suggested that intranasal OXT administration can be combined with behavioral therapies for dogs with behavioral problems related to separation anxiety. Somewhat contrasting this view, van Rooy et al. (2015) in a study examining several candidate genes (OPRM1, AVPR1A, DRD2, OXTR) and their associations with separation-related distress in 42 Australian golden retrievers, found no significant associations between separation-related behavior scores and OXTR gene.

The main purpose of the present study was to explore environmental and genetic influences on dogs' attachment behavior. In order to benefit from the genetic unrelatedness of dogs and their owners, in separate analyses we investigated (1) whether various OXTR polymorphisms of dogs as well as their owners are associated with the attachment behavior of the dogs in two different countries, (2) whether such effects of the dogs' genotypes are affected by the age, sex, and neutering of the dogs. Finally, to tackle potential mechanisms that may mediate the effects of owner genotypes on dog attachment, we analyzed (3) if owner personality, attachment style, and attachment to pets have an effect on dogs' attachment behavior.

## Materials and methods

### Ethics statement

The procedures were approved in accordance with GPS guidelines and national legislation by the Ethical Committees at the University of Veterinary Medicine Vienna and the Medical University of Vienna in Austria (Ref No. 04/12/97/2012 and 2073/2012, respectively) and the University Institutional Animal Care and Use Committee (UIACUC) of Eötvös Loránd University in Hungary (Ref No. XIV-I-001/531-4-2012). Owners of the pet dogs participated in the study on a voluntary basis and gave their consent to the genetic analyses as well as the behavioral testing of their dogs.

### Subjects

Border Collies (*N* = 135; mean age ± *SD*: 4.17 ± 3.01 years, range: 10 months−14 years) kept as pet dogs were recruited in two countries, Austria and Hungary (Austria: male/female: 34/37, neutered/intact: 40/24; Hungary: male/female: 29/35, neutered/intact: 43/19). Dogs and their owners (Austria: male/female: 17/54, mean age ± *SD*: 35.80 ± 10.65 years, age range: 15.99–65.99 years; Hungary: male/female: 7/57, mean age ± *SD*: 34.32 ± 15.18 years, age range: 13.35–54.33 years) participated in the behavioral testing (modified version of the SST test—Horn et al., 2013, see later). Owners and their dogs were recruited for SST on a volunteer basis and there were no specific inclusion/exclusion criteria.

### Experimental set-up

**Figure 1:**
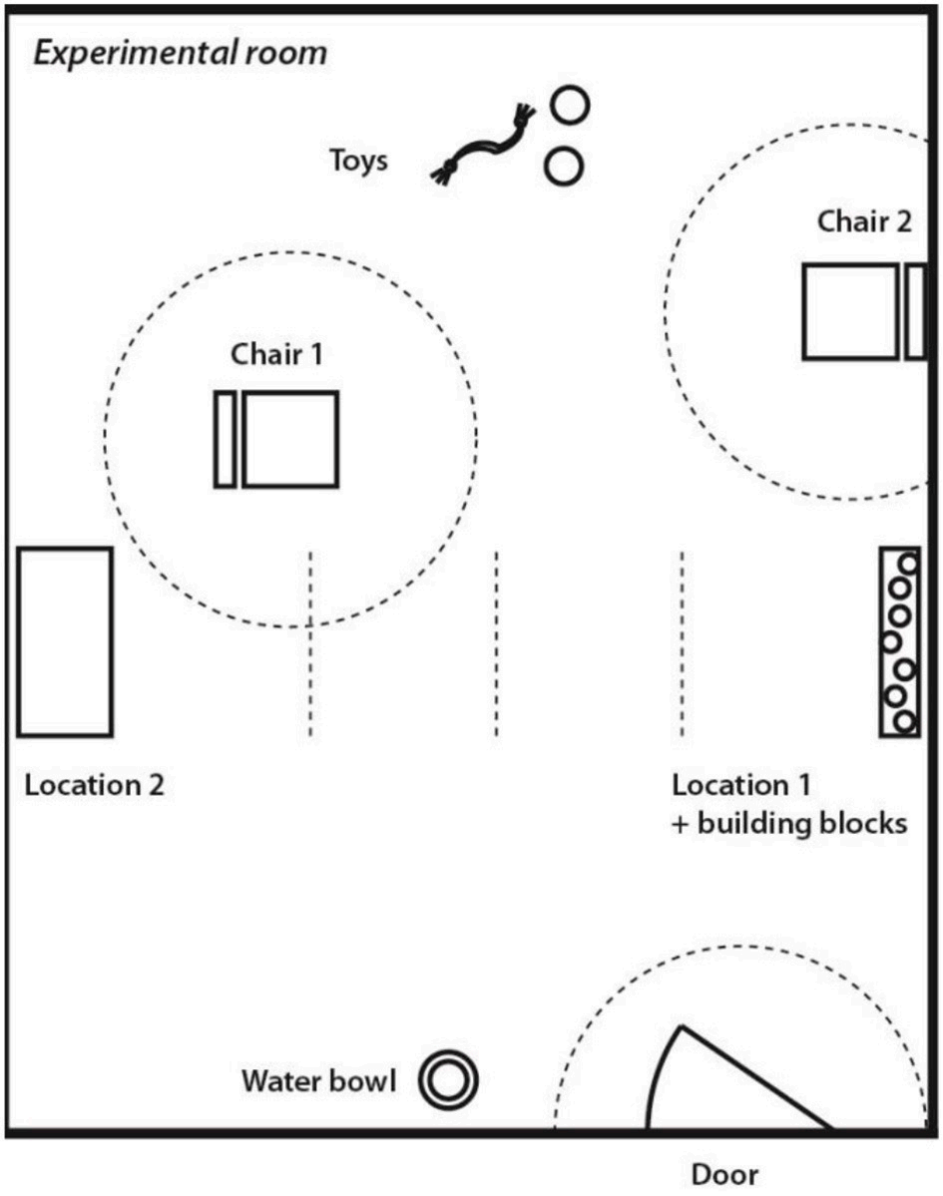
Set-up used in the “Strange Situation Test”.

Dogs' attachment to their human caregivers was tested using the same protocol and experimental set-up in both countries (Horn et al., 2013). Testing took place in an experimental room that was unknown to the dog (5 × 6 m; Figure 1). The experimental room contained four cameras linked to monitoring and recording equipment in an adjacent room. The room contained two chairs (Chair 1, Chair 2), several toys placed on the floor, two elevated locations out of the dog's reach (i.e., windowsill, table; Location 1, Location 2), building blocks placed in Location 1, and a water bowl with fresh water. Three areas with 1 m radius were marked with tape on the floor for later video coding: “close to Chair 1,” “close to Chair 2,” “close to Door.” Additionally, there were three lines indicating the quartiles between the table and the location with the building blocks. The experimental rooms were cleaned with liquid disinfectant to eliminate odors.

### Procedure

Before the start of the experiment, the experimenter explained the procedure in detail to the owner while the dog was sitting in an adjacent room with a helper otherwise uninvolved in the study. The test consisted of seven episodes of ~3 min each. In three episodes a stranger was present in the room. The stranger was of the same gender as the dog's owner and has never been seen by or interacted with the dog prior the experiment.

#### Episode 1 (owner and dog)

The owner entered the experimental room with the dog on leash and sat down on Chair 1. After letting the dog off the leash and placing the leash on the floor next to the chair, the owner first sat quietly and started to fill the questionnaires in without interacting with the dog for 2 min. After that the owner carried building blocks from Location 1 to Location 2 in order to build a tower without interacting with the dog for 1 min. Then the owner sat back on Chair 1 and continued filling the questionnaires.

#### Episode 2 (owner, stranger, and dog + owner leaving)

A stranger entered the room quietly and sat down on Chair 2 opposite of the owner without interacting with the dog for 1 min. Then the stranger got up and initiated play with the dog. After the first minute of the play phase the owner left the room quietly and the stranger continued to play with the dog for another minute.

#### Episode 3 (stranger and dog + stranger leaving)

The stranger returned to Chair 2 and did paperwork without interacting with the dog for 2 min. After that the stranger carried all the building blocks from Location 2 back to Location 1 without interacting with the dog for 1 min. At the end of this phase the stranger left the room quietly.

#### Episode 4 (dog alone)

The dog was left alone in the room for 3 min. This episode was curtailed, if the dog was too distressed by the separation.

#### Episode 5 (owner and dog + owner leaving)

The owner entered the room, paused next to the door without interacting with the dog (~5 s), then greeted the dog shortly (~5 s), and finally sat back on Chair 1. The owner continued filling the questionnaire in without interacting with the dog for 3 min, and at the end of this phase left the room quietly again.

#### Episode 6 (dog alone)

The dog was left alone in the room for 3 min. This episode was curtailed, if the dog was too distressed by the separation.

#### Episode 7 (stranger and dog)

The stranger entered the room, paused next to the door without interacting with the dog (~5 s), then greeted the dog shortly (~5 s), and finally sat back on Chair 2. The stranger continued doing paperwork without interacting with the dog for 3 min and at the end of this phase put the leash on the dog and left the room together with the dog.

### Questionnaires

Owners both from Austria and Hungary were additionally asked to fill in three questionnaires assessing their personality (BFI), romantic relationships (ECR-R), and dog-owner relationship (modified ECR-R).

The 44–item Big Five Inventory (BFI; Supplementary 1) was developed by John and Srivastava (1999). The questionnaire includes 8 questions related to extraversion (e.g., “Is full of energy”); 9 questions for agreeableness (e.g., “Can be cold and aloof”); 9 questions for conscientiousness (e.g., “Tends to be lazy”); 8 questions for neuroticism (e.g., “Is emotionally stable, not easily upset”); and 10 questions for openness (e.g., “Is curious about many different things”). Each item was rated on a Likert scale from 1 (“strongly disagree”) to 5 (“strongly agree”).

The relationship between the owner and his/her partner was measured by the 36-item Experiences in Close Relationship-Revised Questionnaire (ECR-R; Supplementary 2; Fraley et al., 2000). Each item was rated on a Likert scale from 1 (“strongly disagree”) to 7 (“strongly agree”). The questionnaire includes 18 questions related to bond-related anxiety (e.g., “I worry a lot about my relationships”) and 18 questions related for bond-related avoidance (e.g., “I tell my partner just about everything”). The trait scores were calculated by averaging the scores of the variables representing each trait.

The relationship between owner and his/her dog was measured by the modified Experiences in Close Relationship-Revised Questionnaire (ECR-R; Supplementary 3). This questionnaire was developed by Beck and Madresh (2008) based on the 36-item ECR-R for humans (Fraley et al., 2000). Each item was rated on a Likert scale from 1 (“strongly disagree”) to 7 (“strongly agree”). The questionnaire includes 8 questions related to pet-related anxiety (e.g., “My pet makes me feel confident.”) and 8 questions related for pet-related avoidance (e.g., “I prefer not to show a pet how I feel deep down”). The trait scores were calculated by averaging the scores of the variables representing each trait.

Owners were asked to start filling the questionnaires in during the test. As the number of questions in the 3 questionnaires were rather high, they were given the opportunity to finish the questionnaires at home, which lead however to relatively low response rates that varied between 49.6 and 74.8% (owner personality: *N* = 97 in total, Austria: 42, Hungary: 55; romantic relationships: *N* = 101 in total, Austria: 40, Hungary: 61; and dog-owner relationship: *N* = 67 in total, Austria: 34, Hungary: 33).

### DNA-sampling

Before the behavioral test we collected buccal cell samples from those owners who agreed to provide DNA samples (*N* = 66, Austria: 33, Hungary: 33) and from those dogs whose owners agreed to provide genetic information about their dogs (*N* = 130, Austria: 69, Hungary: 61) with a non-invasive method, by swabbing the upper gum area with 4 cotton tips (Wan et al., 2013; Kis et al., 2014). The cotton tips were then sealed in a tube and preserved in the freezer until genotyping (Bence et al., 2016). DNA purification was initiated by incubating the buccal samples at 56°C overnight in 0.2 mg/ml Proteinase K cell lysis buffer. It was followed by protein denaturation using saturated NaCl solution. Finally, DNA was precipitated using isopropanol and ethanol by standard procedures and DNA pellet was resuspended in 100 μl 0.5 × TE (1 × TE: 10 mM Tris pH = 8, 1 mM EDTA) buffer. Typical DNA concentration of the genomic DNA samples isolated from buccal swabs was around 20 ng/μl and measured by NanoDrop® 2000 Spectrophotometer (Thermo Fisher Scientific, Wilmington, Delavare).

### SNP genotyping

−213AG,−94TC and−74CG canine SNPs are located in the 5′ flanking region. The Qiagen Hot-StarTaq polymerase kit (Qiagen, Hilden, Germany) was used for PCR amplification. The reaction mixture contained 1 μM of each primer, approximately 5 ng of DNA template, 200 μM dNTP, 0.025 U HotStarTaq DNA polymerase, 1 × buffer, and 1 × Q-solution supplied together with the enzyme. The PCR cycle consisted of an initial denaturation at 95°C for 15 min, 40 cycles of 1-min denaturation at 95°C, 1-min annealing at various temperatures, a 1-min extension at 72°C, and a 10-min final extension at 72°C. The PCR reaction was performed in a total volume of 10 μl. The −213AG and the −74CG polymorphisms were genotyped by PCR-RFLP method using the primers described in Table 2. PCR products were incubated for 3 h at 37°C in a restriction enzyme mixture containing 0.5 U/μl Hpy99I restriction enzyme (NEB, Ipswich, Massachusetts, USA) for −213 SNP and 0.5 U/μl BsiEI restriction enzyme (NEB, Ipswich, Massachusetts, USA) for −74CG SNP with 1xBSA and 1x NEB4 buffer. Total reaction volume was 16 ml after adding the restriction enzyme mix to the PCR products. The −94TC SNP was genotyped by allele specific amplification (ASA) using the forward and reverse primers described in Table 2. The PCR products were analyzed by conventional submarine agarose gel electrophoresis (Biocenter, Szeged, Hungary), using 2.5% agarose gel and visualized by ethidium bromide staining. Genotype frequencies have been determined and Hardy–Weinberg Equilibrium analyses were carried out. The genotype frequencies were in Hardy–Weinberg equilibrium in both countries. Rare homozygote (AA) genotypes were grouped together with heterozygotes (AA+AG) (Table 1).

**Table 1:** Allele frequencies (%) and number of individuals (N) for Border collies and Owners from Austria and Hungary.

**Dog SNPs**	−**213AG**				−**94TC**				−**74CG**			
Austria	AA	AG	GG	HWE	CC	CT	TT	HWE	CC	CG	GG	HWE
%	0.06	0.22	0.72	*p* = 0.977	0.13	0.58	0.29	*p* = 0.982	0.15	0.27	0.58	*p* = 0.945
N	4	15	49		9	40	20		10	18	38	
Hungary	AA	AG	GG	HWE	CC	CT	TT	HWE	CC	CG	GG	HWE
%	0.13	0.32	0.55	*p* = 0.973	0.44	0.34	0.21	*p* = 0.963	0.24	0.32	0.44	*p* = 0.948
N	8	19	33		27	21	13		14	19	26	
**Owner SNPs**	**rs53576**				**rs1042778**				**rs2254298**			
Austria	CC	CT	TT	HWE	AA	AC	CC	HWE	CC	CT	TT	HWE
%a	0.52	0.22	0.26	*p* = 0.872	0.42	0.42	0.15	*p* = 0.997	0.83	0.17	0.00	*p* = 0.996
N	14	6	7		14	14	5		25	5	0	
Hungary	CC	CT	TT	HWE	AA	AC	CC	HWE	CC	CT	TT	HWE
%	0.39	0.36	0.24	*p* = 0.968	0.13	0.25	0.63	*p* = 0.946	0.84	0.16	0.00	*p* = 0.996
N	13	12	8		4	8	20		26	5	0	

*Statistical tests for Hardy–Weinberg Equilibrium (HWE) are also provided*.

The rs53576 and the rs2254298 polymorphisms were located in intron 3 and the rs1042778 in exon 4 of the human OXTR gene. OXTR rs53576 and rs2254298 SNPs were genotyped by PCR-RFLP method using the primers described in Table 2. PCR products were incubated for 3 h at 37°C in a restriction enzyme mixture containing 0.5 U/μl AvaII restriction enzyme (NEB) for rs53576 SNP and 0.5 U/μl DdeI restriction enzyme (NEB) for rs2254298 SNP, 1x BSA and 1x NEB4 buffer. The PCR products were analyzed by conventional submarine agarose gel electrophoresis (Biocenter, Szeged, Hungary), using 2.5% agarose gel and visualized by ethidium bromide staining.

**Table 2:** Summary of the SNPs included in the analysis.

**Polymorphism/position**	**Primer**	**Sequence (5′-3′)**	**T_A_ (°C)**	**Product size (base pairs)**	**Restriction enzyme**	**Product size (base pairs) after digestion**
**DOG SNPs**						
−213AG/5′ flanking region	Forward Reverse	CCA TTG GAA TCC GCC CCC T CAC CAC CAG GTC GGC TAT G	56 56	635	Hpy99I	C allele: 180+ 201 +254 G allele: 41 +160+180+254
−94TC/5′ flanking region	Forward	CCA TTG GAA TCC GCC CCC T	60	635		
	Reverse	CAC CAC CAG GTC GGC TAT G	60			
	C allele specific	CCG ATC TGC TGG TCC CGG	60	295		
	T allele specific	CCG ATC TGC TGG TCC CGA	60			
−74CG/5′ flanking region	Forward	CCA TTG GAA TCC GCC CCC T	56	635	BsiEI	C allele: 180+201+254 G allele: 41+160+180+254
	Reverse	CAC CAC CAG GTC GGC TAT G	56			
**OWNER SNPs**						
rs53576/intron 3	Forward	ACT GGG GCA ACC AAA CAT CT	56	304	AvaII	G allele: 133 + 61 + 110 A allele: 194 + 110
	Reverse	ACT CTT CAT GGC CCA GAG TG	56			
rs2254298/intron 3	Forward	CTG TCT TTG CAC CTT TGC TA	56	347	DdeI	C allele: 276 + 71 T allele: 246 + 30 + 71
	Reverse	ATG AAA GCA GAG GTT GTG TG	56			
rs1042778/exon 4	Forward	GCT CCA GCC AGA GGA G	60	283		
	Reverse	AGT GGG TTC AGG GTG GTA	60			
	A allele specific	AGC CAC CCC AAG GAG T	60	182		
	C allele specific	AGC CAC CCC AAG GAG G	60			

Genotype frequencies have been determined and Hardy-Weinberg Equilibrium analyses were carried out. The genotype frequencies of any SNPs were in Hardy–Weinberg equilibrium in both country (Austria: *p* = 0.0761, Hungary: *p* = 0.0704). Rare homozygote (AA) genotypes were grouped together with heterozygotes (AA+AG). For detailed information about the SNPs see Table 2.

### Behavior coding

Multivariate analysis of Topál et al. data (1998) (factor and cluster analyses) separated three key aspects of dogs' behavioral structure; Attachment, Anxiety, and Acceptance. In our study we grouped our variables based on these three aspects of the dogs' behavior in the SST. All three composite scores we created were built from several independently coded scores (Table 3). This method of evaluation, in contrast to the previously applied independent behavior variables (e.g., Topál et al., 1998), allowed us to separate the three factors that characterize the dogs' behavior in the SST. By scoring a list of behaviors in different contexts (Table 3) and summing these scores up for each composite score, each dog received a score of Attachment, Anxiety, and Acceptance ranging from −1 to 11.

**Table 3:** Behavior variables observed in Strange Situation Test (D, dog; S, stranger; O, owner).

**Attachment**		**Score**
Owner PRESENT	D is mostly close to O (closest bodypart is within 1 m) when does not explore or play	1
	D does not stand at the door (within 1 m) for more than a few seconds	1
	during the cube-carrying the D mostly watches or follows O	1
	when O first leaves, D follows O to door (within 1 m)	1
	when O leaves the second time, D follows O to door (within 1 m)	1
	when O enters, D approaches (within reaching distance) at once and wags tail	1
Owner ABSENT	D plays with S (at least for 2 s)	−1
	any vocalization	1
	D stands by or orients at door (at least for 2 s−1, most of the time−2)	2
	when S enters, D does not great and tries to sneak out the door	1
	D is mostly at the chair of O (within 1 m) if not at the door	1
**ANXIETY**		
Owner PRESENT	D stands at door (within 1m; at least 2 s−1, most of the time−2)	2
	D does not explore or play at least for 2 s	1
	D positions himself (hides) under/behind O's chair (relative to door or S) for at least 2 s	1
	as soon as O stands up D approaches door within 1 m (before O)	1
	D watches or approaches door while O is carrying cubes (for at least 2 s)	1
	any vocalization (if not clearly asking for the ball)	1
Owner ABSENT	any contact seeking behavior with O before the separation	1
	at 1st separation D vocalizes or runs around up and down or scretches door	1
	at 2nd separation D vocalizes or runs around up and down or scretches door	1
	D follows S to the door when she leaves (within 1 m)	1
	D plays or lies down comfortably (head down) but not at door for at least 2 s	−1
**ACCEPTANCE**		
Owner PRESENT	D approaches S when she 1st enters (at once, within reaching distance)	1
	D gets in physical contact and wags when S 1st enters	1
At any time	D takes toy to S (not during play)	1
	D seeks physical contact (jumps on, snuggles up to, nudges) during the episodes	1
	D avoids S during play (stands off, avoids her touch)	−1
Owner ABSENT	D gets in physical contact and wags when the S enters 2nd time	1
	during cube carrying D mostly watches (1) and also follows (2) S	2
	D plays with S also during separation (at least for 2 s−1, most of the time−2)	2
	D is close (closest bodypart is within 1 m) to S during separation (at least for 2 s−1, most of the time−2)	2

Inter-rater reliability for dogs' behavior was calculated by coding 30% of the sample by four independent coders. Intra-class correlation coefficient (ICC) was used to assess reliability [ICC_(2, 4)_ = 0.976, *p* = 0.002 for Attachment, ICC_(2, 4)_ = 0.923, *p* = 0.018 for Anxiety and ICC_(2, 4)_ = 0.993, *p* < 0.001 for Acceptance].

### Statistical analysis

Three Generalized Estimating Equation models using restricted maximum likelihood estimation were used. The first one (*N* = 66) tested the effects of dog (−213AG,−94TC,−74CG) and owner (rs53576, rs1042778, rs2254298) SNPs, Country (Austria or Hungary) and two-way interactions between dog and owner SNP-s on the behavioral scales. The second model (*N* = 130) tested the effects of dog OXTR SNPs (−213AG,−94TC,−74CG), Country (Austria or Hungary), Dogs' Sex (male or female), Neutering (Intact or Neutered), Age (covariant), as well as all two-way interactions of these with the behavioral scores measured in the SST test (Attachment, Anxiety, Acceptance), except the SNPs' interactions with each other. The third model (*N* = 67) tested the effects of the owners' questionnaire scales (BFI, ECR-R_Partner, ECR-R Dog) on the three behavioral scores of the dogs.Levels of significance (p) were corrected using FDRbh method to adjust for multiple comparisons (see Benjamini and Yekutieli, 2001).

**Figure 2:**
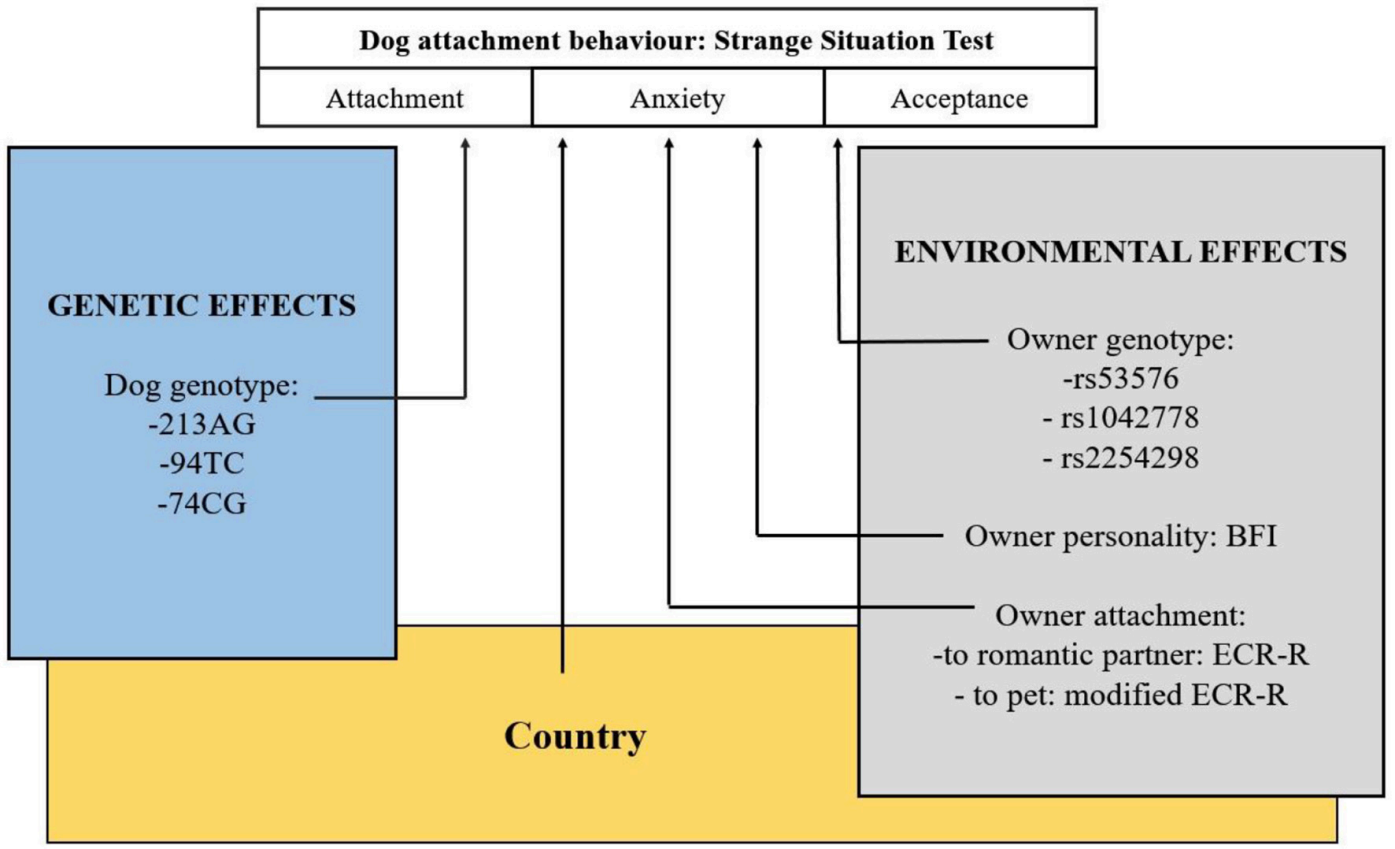
Overview of the study. Examination of environmental and genetic associations of dogs' attachment behavior to their owners. Another study (Kovács et al., 2016b) investigating dogs' social sensitivity has also found significant main effect of the same OXTR polymorphism.

An overview of the study is shown in Figure 2.

## Results

### The interactive effect of dog and owner OXTR polymorphisms (Table 4)

Our analysis showed that dog and owner OXTR SNPs had both main and interactive effects on dog behavior in the SST (Table 4). Attachment composite score was associated with both dog and owner OXTR SNPs (−213AG: *p* < 0.01−74CG: *p* < 0.01, rs1042778: *p* < 0.01, rs2254298: *p* < 0.01, respectively). Interestingly, however, dog and owner OXTR SNPs had also interactive effects (−213AG × rs2254298: *p* < 0.01,−213AG × rs53576: *p* < 0.05,−74CG × rs53576: *p* < 0.01). The effect of Country was confirmed in interaction with dog OXTR SNPs (Country × −213AG: *p* < 0.01, Country × −74CG: *p* < 0.01).

**Table 4:** Summary of the effects of dog and owner OXTR polymorphisms on dogs' Attachment, Anxiety, and Acceptance composite scores as measured in the Strange Situation Test.

**Composite score**	**Effect**	**WCS**	***p***	**Detail**
**Attachment**	**Main effects**			
	−**213AG**	**20.735**	<**0.01**	**AA**+**AG** > **GG**
	−94TC	0.458	>0.1	
	−**74CG**	**16.086**	<**0.01**	**CC**+**CG** > **GG**
	**rs1042778**	**11.573**	<**0.01**	**CC** > **AA**+**AC**
	**rs2254298**	**14.190**	<**0.01**	**CT**+**TT** > **CC**
	rs53576	0.000	>0.1	
	Country	1.887	>0.1	
	**Significant pairwise interactions**			
	−**213AG** × **rs2254298**	**12.340**	<**0.01**	**AA**+**AG (**−**213AG)** +**CC (rs2254298)** > **GG (**−**213AG)** + **CC (rs2254298)**
	−**213AG** × **rs53576**	**7.772**	<**0.05**	**AA**+**AG (**−**213AG)** + **CT**+**TT (rs53576)** > **GG (**−**213AG)** + **CT**+**TT (rs53576)**
	−**74CG** × **rs53576**	**10.904**	<**0.01**	**GG (**−**74CG)** + **CT**+**TT (rs53576)** > **CC**+**CG (**−**74CG)** + **CT**+**TT (rs53576)**
	**Country** × −**213AG**	**15.817**	<**0.01**	**Austria AA**+**AG** > **Austria GG**
	**Country** × −**74CG**	**24.791**	<**0.01**	**Hungary GG** > **Austria GG**
**Anxiety**	**Main effects**			
	−213AG	4.868	>0.1	
	−94TC	0.000	>0.1	
	−**74CG**	**7.808**	<**0.05**	**CC**+**CG** > **GG**
	rs1042778	1.988	>0.1	
	rs2254298	1.369	>0.1	
	rs53576	1.222	>0.1	
	Country	3.954		
	**Significant pairwise interactions**			
	−**213AG** × **rs53576**	**14.826**	<**0.01**	**AA**+**AG (**−**213AG)** + **CT**+**TT (s53576)** > **AA**+**AG (**−**213AG)** + **CC (rs53576)**
	−**74CG** × **rs53576**	**5.955**	<**0.05**	**GG (**−**74CG)** + **CT**+**TT (rs53576)** > **CC**+**CG (**−**74CG)** + **CT**+**TT (rs53576)**
	**Country** × −**213AG**	**20.088**	<**0.01**	**Austria AA**+**AG** > **Austria GG**
	**Country** × −**74TC**	**10.421**	<**0.01**	**Hungary GG** > **Austria GG**
**Acceptance**	**Main effects**			
	−213AG	4.562	>0.1	
	−94TC	0.101	>0.1	
	−**74CG**	**6.233**	<**0.05**	**CC**+**CG** > **GG**
	rs1042778	0.548	>0.1	
	rs2254298	1.480	>0.1	
	**rs53576**	**6.331**	<**0.05**	**CT**+**TT** > **CC**
	Country	1.592	>0.1	
	**Significant pairwise interactions**			
	−**213AG** × **rs1042778**	**13.561**	<**0.01**	**GG (**−**213AG)** + **CT**+**TT (rs53576)** > **GG (**−**213AG)** + **CC (rs53576)**
	−**213AG** × **rs53576**	**12.347**	<**0.01**	**CC (**−**94TC)** + **CT**+**TT (rs53576)** > **CT**+**TT (**−**94TC)** + **CT**+**TT (rs53576)**
	−**94TC** × **rs1042778**	**12.695**	<**0.01**	**CT**+**TT (**−**94TC)** + **CC (rs1042778)** > **CC (**−**94TC)** + **CC (rs1042778)**
	−**74CG** × **rs1042778**	**12.018**	<**0.01**	**CC**+**CG (**−**74CG)** + **AA**+**AC (rs1042778)** > **GG (**−**74CG)** + **AA**+**AC (rs1042778)**
	−**74CG** × **rs53576**	**16.541**	<**0.01**	**CC**+**CG (**−**74CG)** + **CT**+**TT (rs53576)** > **GG (**−**74CG)** + **CT**+**TT (rs53576)**

*Significant effects are highlighted in bold*.

The same holds true for the Anxiety score. Apart from the main effect of dog −74CG SNP (*p* < 0.05), interactive effects of the dog and human OXTR genotypes were also found (-213AG × rs53576: *p* < 0.01,−74CG × rs53576: *p* < 0.05). We also found a significant interactions between Country and dog OXTR SNPs (Country × −213AG, Country × −74CG, both *p* < 0.01).

Similarly, Acceptance of the stranger was also associated with the OXTR polymorphisms in dogs (−74CG: *p* < 0.05) and in the owners (rs53576, *p* < 0.05), and there were also significant interactions between dog- and owner OXTR SNPs (-213AG × rs1042778: *p* < 0.01,−213AG × rs53576: *p* < 0.01,−94TC × rs1042778: *p* < 0.01,−74CG × rs1042778: *p* < 0.01,−74CG × rs53576: *p* < 0.01).

### The effect of dog OXTR polymorphisms and dog characteristics (Table 5)

Two OXTR polymorphisms (−213AG,−74CG) and other dog characteristics (such as country, age, sex, and neuter status) have been found to influence the dogs' behavior in the SST as a main effect or in interaction with each other. Attachment was most notably associated with Country (*p* < 0.01) and Country × Neuter status interaction was also significant (*p* < 0.05). Anxiety was associated with the −213AG SNP (*p* < 0.05) and Country (*p* < 0.05). There were significant interactions between Sex and dog OXTR SNPs (Sex × −213AG: *p* < 0.05; Sex × −74CG: *p* < 0.05) and Neuter status × −213AG was also significant (*p* < 0.05). The analysis of the Acceptance of the stranger showed no main effects of OXTR SNPs, but the Neuter status (*p* < 0.05) and the interaction between Neuter status and Age (*p* < 0.05) were significant.

**Table 5:** The effects of dog OXTR polymorphisms and dog characteristics on Attachment, Anxiety, and Acceptance composite scores as measured in the Strange Situation Test.

**Composite score**	**Effect**	**WCS**	***p***	**Details**
**Attachment**	**Main effects**			
	−213AG	4.854	>0.1	
	−94TC	0.000	>0.1	
	74CG	1.325	>0.1	
	**Country**	**16.868**	<**0.01**	**Hungary** > **Austria**
	Age	4.207	>0.1	
	Sex	1.762	>0.1	
	Neuter status	0.137	>0.1	
	**Significant pairwise interactions**			
	**Country** × **Neuter status**	**8.486**	<**0.05**	**Neutered Hungary** > **Neutered Austria**
**Anxiety**	**Main effects**			
	−**213AG**	**12.349**	<**0.05**	**AA**+**AG** > **GG**
	−94TC	0.099	>0.1	
	−74CG	4.105	>0.1	
	**Country**	**10.516**	<**0.05**	**Hungary** > **Austria**
	Age	0.228	>0.1	
	Sex	3.498	>0.05	
	Neuter status	0.619	>0.1	
	**Significant pairwise interactions**			
	**Sex** × −**213AG**	**11.027**	<**0.05**	**Male AA**+**AG** > **Male GG**
	**Sex** × −**74CG**	**7.723**	<**0.05**	**Female CC**+**CG** > **Female GG**
	**Neuter status** × −**213AG**	**8.975**	<**0.05**	**Neutered AA**+**AG** > **Neutered GG**
**Acceptance**	**Main effects**			
	−213AG	0.179	> 0.1	
	−94TC	1.469	> 0.1	
	−74CG	1.161	>0.1	
	Country	0.055	>0.1	
	Age	0.167	>0.1	
	Sex	3.098	>0.05	
	**Neuter status**	**8.411**	<**0.05**	**Neutered** > **Intact**
	**Significant pairwise interactions**			
	**Neuter status** × **Age**	**11.880**	<**0.05**	**Intact young** > **Neutered young**

*Significant effects are highlighted in bold*.

### The effects of owner personality and relationship experiences (Table 6, Supplementary 4)

Our analysis indicates that dogs' behavior in the SST is related to several aspects of owner personality and to the owner's experience with romantic partners and dogs. The dogs' Attachment score was significantly associated with their owners' relationship both with their romantic partners and their dogs. Namely, higher Attachment scores in dogs were associated with lower Bond-related avoidance (*p* < 0.01) and higher Pet-related avoidance (*p* < 0.01) of their owners.

**Table 6:** The effects of owner personality and relationship experiences with both romantic partners and dogs on dogs' Attachment, Anxiety, and Acceptance composite scores as measured in the Strange Situation Test.

**Composite score**	**Effect**	**WCS**	***p***	**Detail**
**Attachment**	Extraversion	3.545	>0.05	
	Agreeableness	0.044	>0.1	
	Conscientiousness	1.671	>0.1	
	Neuroticism	0.525	>0.1	
	Openness	1.430	>0.1	
	Bond-related anxiety	0.545	>0.1	
	**Bond-related avoidance**	**30.691**	<**0.01**	**lower Bond-related avoidance** > **higher Bond-related avoidance**
	**Pet-related avoidance**	**18.539**	<**0.01**	**higher Pet-related avoidance** > **lower Pet-related avoidance**
	Pet-related anxiety	3.601	>0.01	
**Anxiety**	**Extraversion**	**4.990**	<**0.05**	**higher Extraversion** > **lower Extraversion**
	Agreeableness	0.001	>0.1	
	Conscientiousness	4.150	>0.1	
	Neuroticism	1.525	>0.1	
	**Openness**	**9.577**	<**0.01**	**higher Openness** > **lower Openness**
	**Bond-related anxiety**	**8.944**	<**0.01**	**higher Bond-related anxiety** > **lower Bond-related anxiety**
	**Bond-related avoidance**	**15.345**	<**0.01**	**lower Bond-related Avoidance** > **higher Bond-related Avoidance**
	**Pet-related avoidance**	**46.042**	<**0.01**	**higher Pet-related avoidance** > **lower Pet-related avoidance**
	**Pet-related anxiety**	**18.790**	<**0.01**	**higher Pet-related anxiety** > **lower Pet-related anxiety**
**Acceptance**	Extraversion	0.896	>0.1	
	Agreeableness	0.393	>0.1	
	Conscientiousness	2.837	>0.05	
	Neuroticism	0.604	>0.1	
	**Openness**	**11.588**	<**0.01**	**lower Openness** > **higher Openness**
	Bond-related anxiety	0.396	>0.1	
	**Bond-related avoidance**	**16.032**	<**0.01**	**higher Pet-related avoidance** > **lower Pet-related avoidance**
	**Pet-related avoidance**	**40.075**	<**0.01**	**higher Pet-related avoidance** > **lower Pet-related avoidance**
	**Pet-related anxiety**	**20.356**	<**0.01**	**higher Pet-related anxiety** > **lower Pet-related anxiety**

*Significant effects are highlighted in bold*.

Higher Anxiety scores in dogs were in association with higher Extraversion (*p* < 0.05) and Openness (*p* < 0.01), as well as with higher Bond-related anxiety (*p* < 0.01), Pet-related anxiety (*p* < 0.01), and Pet-related avoidance scores (*p* < 0.01), and lower Bond-related avoidance (*p* < 0.01) of their owners.

**Figure 3:**
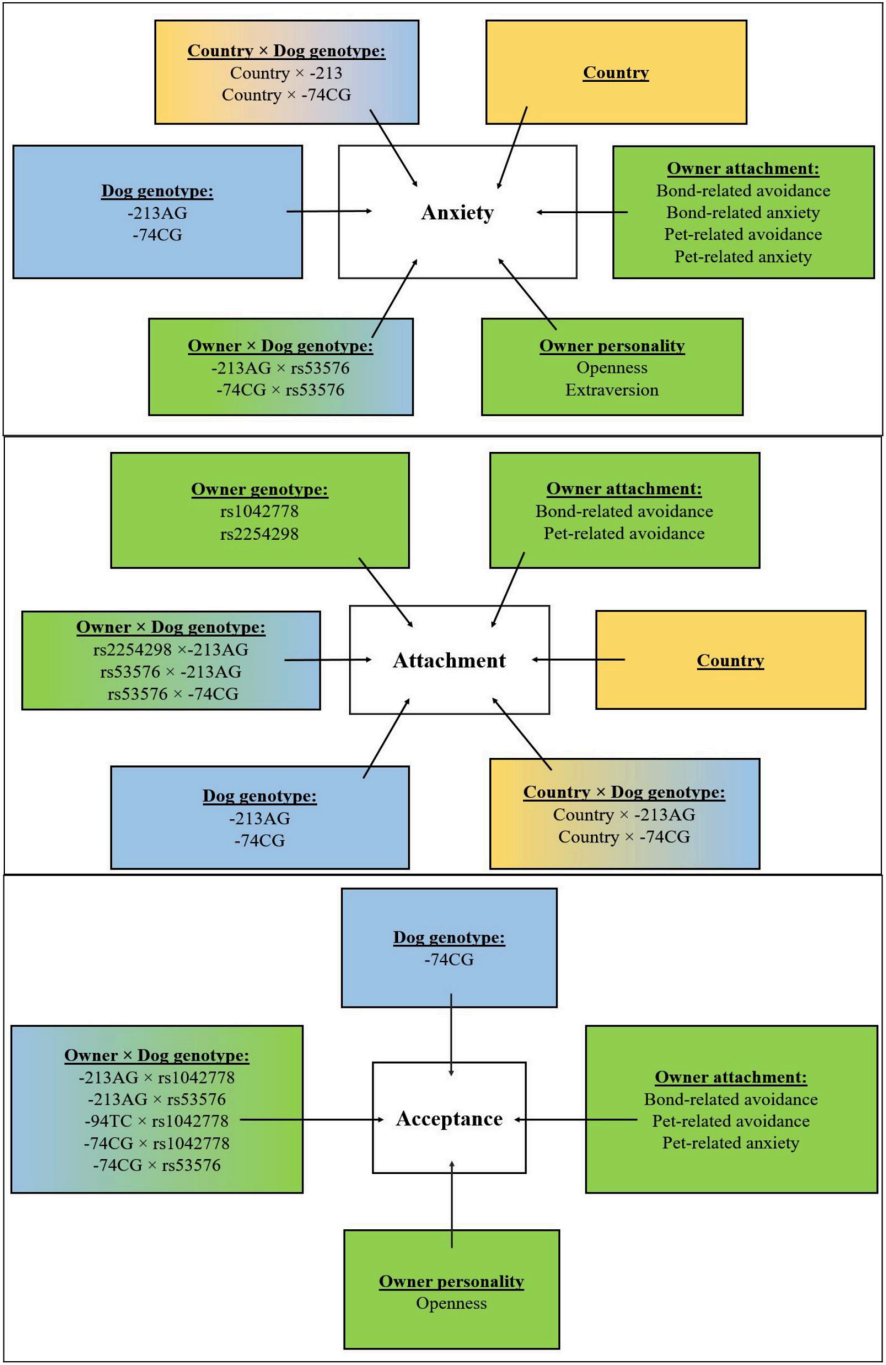
Overview of the results. Effects of country (yellow boxes), environmental (green boxes), and genetic (blue boxes) associations of dogs' behavior (attachment, anxiety, and acceptance composite scores) and their interactions.

As regards Acceptance, higher scores in dogs were in association with lower Openness (*p* < 0.01) and higher Bond-related avoidance (*p* < 0.01), Pet-related anxiety (*p* < 0.01), and Pet-related avoidance scores (*p* < 0.01) of their owners. See Figure 3 for the overview of the results.

## Discussion

Chen et al. (2011) had suggested that one source of the variation in human infants' attachment to their mothers is the polymorphism of their OXTR gene but they remained cautious about this conclusion due to the genetic relatedness of infants and their parents. Based on the analogy between infant-mother and dog-owner attachment, our findings seem to confirm their suggestion, as the present study provides the first evidence that genetic variations in dogs' OXTR gene are associated with their attachment behavior to their owners. All behavioral aspects measured in the SST (Attachment, Anxiety, and Acceptance) showed significant association with all three dog OXTR SNPs investigated in this study (as a main or an interaction effect). All 3 canine OXTR SNPs have proved to have behavioral associations also in former studies. Behavioral associations of the −213AG polymorphism have already been reported in other studies. The −213AG polymorphism had been shown to be associated with proximity seeking not only in Border Collies but also in German Shepherds (Kis et al., 2014). Another study (Kovács et al., 2016b) investigating dogs' social sensitivity has also found significant main effect of the same OXTR polymorphism (−213AG) on readiness to look at the human face in two test situations. Moreover, Turcsán (2017) found that this SNP was associated with greeting behavior, especially when the dog had no prior negative experience with the experimenter. Oláh et al. (2017) also found that dogs' first reaction and their friendliness in the threatening approach test were significantly modulated by −213AG and −74GC polymorphisms. In line with our finding that −213AG polymorphism is associated with Stranger acceptance, Romero et al. (2014) also found that oxytocin modulates social motivation to approach and affiliate with conspecifics and human partners. Earlier studies (Windle et al., 1997; Neumann, 2002; Bello et al., 2008) had also shown, that oxytocin can regulate the activity of the hypothalamic–pituitary–adrenal axis, thereby modifying the stress response. This mechanism may explain the associations we found between the OXTR polymorphisms and Anxiety composite score.

We found some differences between the results of different models. For example −74CG has significant main effects on dogs' Attachment, Anxiety as well as Acceptance scores in our first model (when testing its effects in interaction with the owners' genotypes), but in the second model it has an influence only on dogs' Anxiety and only when tested in interaction with dogs' sex. Moreover, while −74CG (and not −213AG) has a main effect on dogs' Anxiety in the first model,−213AG (and not −74CG) has a main effect on the Anxiety in our second model. This seems to result from the facts that sex of the dogs influenced the associations of two canine OXTR SNPs, namely the effects of −213AG and −74CG on dogs' Anxiety. It has been shown both in humans (e.g., Herzmann et al., 2013) and dogs (Nagasawa et al., 2015; Kovács et al., 2016a; Turcsán, 2017) that oxytocin can have differential effects on males and females. In prairie voles more is known about the underlying mechanisms of such sex differences, as Smeltzer et al. (2006) found higher binding by the oxytocin receptors in the medial prefrontal cortex in female than in male prairie voles. Another possible explanation is that steroid hormones, such as estradiol and progesterone, can modulate the affinity of the oxytocin receptors (estradiol enhances OXT receptor affinity, while progesterone has been shown to decrease receptor binding; Gimpl et al., 2002; Choleris et al., 2008).

Another important finding of the study is that both dog and owner OXT genetic variation shapes the dog-owner attachment in an interactive manner. Earlier research has also shown a mutual effect of both dogs and their owners on the peripheral oxytocin levels of both parties (Nagasawa et al., 2009, 2015). This is the first study, however, providing evidence that the oxytocin system of both parties impacts on dogs' attachment behavior. We found significant effects of two human OXTR SNPs (rs2254298 and rs1042778) on the Attachment composite score and one SNP (rs53576) on Acceptance, and several significant interactions of the effects of the human and dog OXTR gene on the attachment behavior of dogs (e.g., −213AG × rs2254298,−74CG × rs53576,−94TC × rs1042778,−74CG × rs1042778). Dog owners' behavior is likely to be one of the greatest environmental factors influencing the dogs' attachment behavior. It is possible that the owners' behavior mediates this link between the owners' genotype and the dogs' relationship to them, particularly given Bakermans-Kranenburg's and van Ijzendoorn (2008) finding that OXTR is related to parenting style in humans. As dogs are unrelated to their human caregivers, the owner's genetic background may have an influence on their parenting style or other relevant behavior that, in turn, through epigenetic processes, affects the dogs' attachment behavior or the effects of the dogs' own OXTR genotype on it. However, given that in this study a considerable number of human DNA samples were missing, future studies with larger sample sizes need to consolidate these results.

As a further confirmation of owner influences on dog attachment, we have also found that the owners' Bond-related avoidance to their partners and Pet-related avoidance influenced all the three composite scores of their dogs' attachment, Openness and Pet-related anxiety affected dogs' Anxiety and Acceptance, while Extraversion personality trait influenced the Anxiety composite score. Previously, Konok et al. (2015) examined in a questionnaire study whether owners' attachment style and personality traits influence the occurrence of separation-relation disorder in the dogs. They found that owners scoring higher on self-reported attachment avoidance are more likely to have dogs with separation-related disorder. They suggested that owners' attachment style influences their caregiving behavior toward their dogs, and owners with attachment avoidance may show less consistent responsiveness to their dog's needs. Schöberl et al. (2016) also investigated the effects of owner personality and found that owners with high neuroticism and agreeableness had dogs with lower cortisol reactivity in the Strange Situation Test. In contrast, in humans, children's insecure attachment, behavior problems and separation anxiety disorders are often associated with mothers' neuroticism and anxiety disorder (Manassis et al., 1994; Biederman et al., 2001; Kochanska et al., 2004). Interestingly, in our study the Neuroticism personality trait associated with none of the dog composite scores. Our finding about the correlation between owners' personality and their dogs' behavior is especially important from an applied perspective, as environmental factors may have the potential to modify oxytocin-related behavioral changes.

Finally, one of the most powerful effects we found was a difference between Austria and Hungary. There were main effects of country on two of the three behavioral components (Attachment and Anxiety composite scores) with dogs in Hungary showing higher Attachment and Anxiety, than dogs in Austria. Country has also influenced the effect of dog genetic background on attachment; similarly to Chen's et al. (2011) study where a certain OXTR genotype was associated with secure attachment in a non-Caucasian sample but not in a Caucasian sample. Oxytocin functions, similarly to serotonin (Yoshida et al., 2009) and dopamine functions (Liu and Wang, 2003), are likely be influenced by multiple factors, including other genetic polymorphisms that vary across ethnic populations (e.g., Chang et al., 1996; Kunugi et al., 1997). These uncontrolled genetic differences in our study could potentially mask the function of oxytocin and thus may have weakened the measurable association between variations in OXTR function and attachment behavior of dogs. Alternatively or additionally, different components of the dogs' social environment may differ between the two countries. It has been suggested that the behavioral expression of certain genotypes is sensitive to input from the social environment (Way and Taylor, 2010). Kim et al. (2010) suggested that the social environment can alter or even reverse the phenotypic expression of different genotypes. They found that culture-specific norms as a form of social input can also affect phenotypic expression of OXTR.

In conclusion, our study provides experimental evidence that genetic variations of the OXTR gene in both dogs and their owners, as well as various aspects of dogs' environmental background are associated with their attachment to their human caregivers. Based on previous and present results we propose that polymorphism in the oxytocin receptor gene is a potentially important factor in regulating dog-human relationship. Although the complex joint effects of genetic and environmental factors on dogs' human-directed social behavior warrant further investigation, these findings offer a promising approach to studying causes and treatments of separation anxiety in dogs.

## Author contributions

KK, ZV, AK, and JT: Designed the study. All authors prepared the study material and data acquisition. KK and DK: Entered the data and prepared it for statistical analyses; KK, ZV, AK, BT, and JT: Analyzed the data; KK, ZV, and JT: Interpreted the data; ZV, ZR, and JT: Obtained funding; KK: Wrote the first draft of the manuscript; KK, ZV, AK, MG, and JT: Critically revised the manuscript for important intellectual content. All authors gave final approval of the manuscript version to be published and agreed to be accountable for all aspects of the work in ensuring that questions related to the accuracy or integrity of any part of the work are appropriately investigated and resolved.

### Conflict of interest statement

The authors declare that the research was conducted in the absence of any commercial or financial relationships that could be construed as a potential conflict of interest.

## References

[B1] Ainsworth M. D. Blehar D. S. Waters E. Wall S. (1978). Patterns of Attachment: A Psychological Study of the Strange Situation. Oxford, UK: Lawrence Erlbaum.

[B2] Arahori M. Hori Y. Saito A. Chijiiwa H. Takagi S. Ito Y. (2016). The oxytocin receptor gene (OXTR) polymorphism in cats (*Felis catus*) is associated with “Roughness” assessed by owners. J. Vet. Behav. Clin. Appl. Res. 11, 109–112. 10.1016/j.jveb.2015.07.039

[B3] Bakermans-Kranenburg M. J. van Ijzendoorn M. H. (2008). Oxytocin receptor (OXTR) and serotonin transporter (5-HTT) genes associated with observed parenting. Soc. Cogn. Affect. Neurosci. 3, 128–134. 10.1093/scan/nsn004 19015103 PMC2555463

[B4] Barry R. A. Kochanska G. Philibert R. A. (2008). G x E interaction in the organization of attachment: mothers' responsiveness as a moderator of children's genotypes. J. Child Psychol. Psychiatry. 49, 1313–1320. 10.1111/j.1469-7610.2008.01935.x 19120710 PMC2688730

[B5] Beck L. Madresh E. A. (2008). Romantic partners and four-legged friends: an extension of attachment theory to relationships with pets. Anthrozoos 21, 43–56. 10.2752/089279308X274056

[B6] Bello D. White-Traut R. Schwertz D. Pournajafi-Nazarloo H. Carter C. S. (2008). An exploratory study of neurohormonal responses of healthy men to massage. J. Altern. Complement. Med. 14, 387–394. 10.1089/acm.2007.0660 18576922

[B7] Bence M. Marx P. Szantai E. Kubinyi E. Ronai Z. Banlaki Z. (2016). Lessons from the canine Oxtr gene: populations, variants and functional aspects. Genes Brain Behav. 16, 427–438. 10.1111/gbb.12356 27860243

[B8] Benjamini Y. Yekutieli D. (2001). The control of the false discovery rate in multiple testing under dependency. Ann. Stat. 29, 1165–1188. 10.1214/aos/1013699998

[B9] Biederman J. Faraone S. V. Hirshfeld-Becker D. R. Friedman D. Robin J. A. Rosenbaum J. F. (2001). Patterns of psychopathology and dysfunction in high-risk children of parents with panic disorder and major depression. Am. J. Psychiatry 158, 49–57. 10.1176/appi.ajp.158.1.49 11136633

[B10] Bowlby J. (1958). The nature of the child' s tie to his mother. Int. J. Psychoanal. 39, 350–373. 13610508

[B11] Bowlby J. (1969). Attachment and loss: volume I: attachment. Int. Psycho-Anal. Libr. 79, 1–401.

[B12] Brennan K. A. Clark C. L. Shaver P. R. (1998). Self-report measurement of adult romantic attachment: an integrative overview, in Attachment Theory and Close Relationships, eds Simpson J. A. Rholes W. S. (New York, NY: Guilford Press), 46–76.

[B13] Chang F. M. Kidd J. R. Livak K. J. Pakstis A. J. Kidd K. K. (1996). The world-wide distribution of allele frequencies at the human dopamine D4 receptor locus. Hum. Genet. 98, 91–101. 10.1007/s004390050166 8682515

[B14] Chen F. S. Barth M. E. Johnson S. L. Gotlib I. H. Johnson S. C. (2011). Oxytocin receptor (OXTR) polymorphisms and attachment in human infants. Front. Psychol. 2:200. 10.3389/fpsyg.2011.00200 21904531 PMC3161247

[B15] Choleris E. Devidze N. Kavaliers M. Pfaff D. W. (2008). Steroidal/neuropeptide interactions in hypothalamus and amygdala related to social anxiety. Prog. Brain Res. 170, 291–303. 10.1016/S0079-6123(08)00424-X 18655890

[B16] Cimarelli G. Virányi Z. Turcsán B. Rónai Z. Sasvári-Székely M. Bánlaki Z. (2017). Social behavior of pet dogs is associated with peripheral OXTR methylation. Front. Psychol. 8:549. 10.3389/fpsyg.2017.00549 28443051 PMC5385375

[B17] Clutton-Brock J. (1999). A Natural History of Domesticated Mammals. Cambridge, UK: Cambridge University Press.

[B18] Council N. Relations F. Small S. A. (1988). Parental self-esteem and its relationship to childrearing practices, parent-adolescent interaction, and adolescent behavior. J. Marriage Fam. 50, 1063–1072. 10.2307/352115

[B19] Diamond L. M. (2001). Contributions of psychophysiology to research on adult attachment: review and recommendations. Pers. Soc. Psychol. Rev. 5, 276–295. 10.1207/S15327957PSPR0504_1

[B20] Fearon P. Shmueli-Goetz Y. Viding E. Fonagy P. Plomin R. (2014). Genetic and environmental influences on adolescent attachment. J. Child Psychol. Psychiatry Allied Discip. 55, 1033–1041. 10.1111/jcpp.12171 24256475 PMC4366883

[B21] Fonagy P. (2001). The human genome and the representational world: the role of early mother-infant interaction in creating an interpersonal interpretive mechanism. Bull. Menninger Clin. 65, 427–448. 10.1521/bumc.65.3.427.19844 11531137

[B22] Fraley R. C. Spieker S. J. (2003). Are infant attachment patterns continuously or categorically distributed? A taxometric analysis of strange situation behavior. Dev. Psychol. 39, 387–404. 10.1037/0012-1649.39.3.387 12760508

[B23] Fraley R. C. Waller N. G. Brennan K. A. (2000). An item response theory analysis of self-report measures of adult attachment. J. Pers. Soc. Psychol. 78, 350–365. 10.1037/0022-3514.78.2.350 10707340

[B24] Gácsi M. Topál J. Miklósi A. Dóka A. Csányi V. (2001). Attachment behavior of adult dogs (*Canis familiaris*) living at rescue centers: forming new bonds. J. Comp. Psychol. 115, 423–431. 10.1037/0735-7036.115.4.423 11824906

[B25] Gillath O. Bunge S. A. Shaver P. R. Wendelken C. Mikulincer M. (2005). Attachment-style differences in the ability to suppress negative thoughts: exploring the neural correlates. Neuroimage 28, 835–847. 10.1016/j.neuroimage.2005.06.048 16087352

[B26] Gillath O. Shaver P. R. Baek J.-M. Chun D. S. (2008). Genetic correlates of adult attachment style. Pers. Soc. Psychol. Bull. 34, 1396–1405. 10.1177/0146167208321484 18687882

[B27] Gimpl G. Wiegand V. Burger K. Fahrenholz F. (2002). Cholesterol and steroid hormones: modulators of oxytocin receptor function. Prog. Brain Res. 139, 43–55. 10.1016/S0079-6123(02)39006-X 12436925

[B28] Haram M. Tesli M. Dieset I. Steen N. E. Røssberg J. I. Djurovic S.. (2014). An attempt to identify single nucleotide polymorphisms contributing to possible relationships between personality traits and oxytocin-related genes. Neuropsychobiology 69, 25–30. 10.1159/000356965 24458227

[B29] Herzmann G. Bird C. W. Freeman M. Curran T. (2013). Effects of oxytocin on behavioral and ERP measures of recognition memory for own-race and other-race faces in women and men. Psychoneuroendocrinology 38, 2140–2151. 10.1016/j.psyneuen.2013.04.002 23648370 PMC3775862

[B30] Horn L. Huber L. Range F. (2013). The importance of the secure base effect for domestic dogs - evidence from a manipulative problem-solving task. PLoS ONE 8:e65296. 10.1371/journal.pone.0065296 23734243 PMC3667003

[B31] John O. P. Srivastava S. (1999). The Big Five trait taxonomy: history, measurement, and theoretical perspectives. Handb. Pers. Theory Res. 2, 102–138.

[B32] Kendler K. S. Sham P. C. MacLean C. J. (1997). The determinants of parenting: an epidemiological, multi-informant, retrospective study. Psychol. Med. 27, 549–563. 10.1017/S0033291797004704 9153676

[B33] Kim H. S. Sherman D. K. Sasaki J. Y. Xu J. Chu T. Q. Ryu C.. (2010). Culture, distress, and oxytocin receptor polymorphism (OXTR) interact to influence emotional support seeking. Proc. Natl. Acad. Sci. U.S.A. 107, 15717–15721. 10.1073/pnas.1010830107 20724662 PMC2936623

[B34] Kis A. Bence M. Lakatos G. Pergel E. Turcsán B. Pluijmakers J.. (2014). Oxytocin receptor gene polymorphisms are associated with human directed social behavior in dogs (*Canis familiaris*). PLoS ONE 9:e83993. 10.1371/journal.pone.0083993 24454713 PMC3893090

[B35] Kochanska G. Friesenborg A. E. Lange L. A. Martel M. M. Kochanska G. (2004). Parents' personality and infants' temperament as contributors to their emerging relationship. J. Pers. Soc. Psychol. 86, 744–759. 10.1037/0022-3514.86.5.744 15161398

[B36] Konok V. Kosztolányi A. Rainer W. Mutschler B. Halsband U. Miklósi Á. (2015). Influence of owners' attachment style and personality on their dogs' (*Canis familiaris*) separation-related disorder. PLoS ONE 10:e0118375. 10.1371/journal.pone.0118375 25706147 PMC4338184

[B37] Kovács K. Kis A. Kanizsár O. Hernádi A. Gácsi M. Topál J. (2016a). The effect of oxytocin on biological motion perception in dogs (*Canis familiaris*). Anim. Cogn. 19, 513–522. 10.1007/s10071-015-0951-4 26742930

[B38] Kovács K. Kis A. Pogány Á. Koller D. Topál J. (2016b). Differential effects of oxytocin on social sensitivity in two distinct breeds of dogs (*Canis familiaris*). Psychoneuroendocrinology 74, 212–220. 10.1016/j.psyneuen.2016.09.010 27665081

[B39] Kunugi H. Hattori M. Kato T. Tatsumi M. Sakai T. Sasaki T.. (1997). Serotonin transporter gene polymorphisms: ethnic difference and possible association with bipolar affective disorder. Mol. Psychiatry 2, 457–462. 10.1038/sj.mp.4000334 9399688

[B40] Lakatos K. Toth I. Nemoda Z. Ney K. Sasvari-Szekely M. Gervai J. (2000). Dopamine D4 receptor (DRD4) gene polymorphism is associated with attachment disorganization in infants. Mol. Psychiatry 5, 633–637. 10.1038/sj.mp.4000773 11126393

[B41] Liu Y. Wang Z. (2003). Nucleus accumbens oxytocin and dopamine interact to regulate pair bond formation in female prairie voles. Neuroscience 121, 537–544. 10.1016/S0306-4522(03)00555-4 14568015

[B42] Lucht M. J. Barnow S. Sonnenfeld C. Rosenberger A. Grabe H. J. Schroeder W.. (2009). Associations between the oxytocin receptor gene (OXTR) and affect, loneliness and intelligence in normal subjects. Prog. Neuropsychopharmacol. Biol. Psychiatry 33, 860–866. 10.1016/j.pnpbp.2009.04.004 19376182

[B43] Manassis K. Bradley S. Goldberg S. Hood J. Swinson R. P. (1994). Attachment in mothers with anxiety disorders and their children. J. Am. Acad. Child Adolesc. Psychiatry 33, 1106–1113. 10.1097/00004583-199410000-00006 7982861

[B44] Mariti C. Carlone B. Ricci E. Sighieri C. Gazzano A. (2014). Intraspecific attachment in adult domestic dogs (*Canis familiaris*): preliminary results. Appl. Anim. Behav. Sci. 152, 64–72. 10.1016/j.applanim.2013.12.002

[B45] Metsäpelto R. L. Pulkkinen L. (2003). Personality traits and parenting: neuroticism, extraversion, and openness to experience as discriminative factors. Eur. J. Pers. 17, 59–78. 10.1002/per.468

[B46] Mikulincer M. Shaver P. R. Pereg D. (2003). Attachment theory and affect regulation: the dynamics, development, and cognitive consequences of attachment-related strategies. Motiv. Emot. 27, 77–102. 10.1023/A:1024515519160

[B47] Naderi S. Miklósi A. Dóka A. Csányi V. (2002). Does dog-human attachment affect their inter-specific cooperation? Acta Biol. Hung. 53, 537–550. 10.1556/ABiol.53.2002.4.13 12501937

[B48] Nagasawa M. Kikusui T. Onaka T. Ohta M. (2009). Dog's gaze at its owner increases owner's urinary oxytocin during social interaction. Horm. Behav. 55, 434–441. 10.1016/j.yhbeh.2008.12.002 19124024

[B49] Nagasawa M. Mitsui S. En S. Ohatani N. Ohta M. Sakuma Y.. (2015). Oxytocin-gaze positive loop and the coevolution of human-dog bonds. Science 348, 333–336. 10.1126/science.1261022 25883356

[B50] Neumann I. D. (2002). Involvement of the brain oxytocin system in stress coping: interactions with the hypothalamo-pituitary-adrenal axis. Prog. Brain Res. 139, 147–162. 10.1016/S0079-6123(02)39014-9 12436933

[B51] Oláh K. Topál J. Kovács K. Kis A. Koller D. Park S. J. (2017). Gaze-following and reaction to an aversive social interaction have corresponding associations with variation in the OXTR gene in dogs but not in human infants. Front. Psychol. Psychol. 8:2156 10.3389/fpsyg.2017.02156 PMC5732940 29312041

[B52] Prato-Previde E. Custance D. Spiezio C. Sabatini F. (2003). Is the dog-human relationship an attachment bond? An observational study using Ainsworth's Strange Situation. Behaviour 140, 225–254. 10.1163/156853903321671514

[B53] Romero T. Nagasawa M. Mogi K. Hasegawa T. Kikusui T. (2014). Oxytocin promotes social bonding in dogs. Proc. Natl. Acad. Sci. U.S.A. 111, 9085–9090. 10.1073/pnas.1322868111 24927552 PMC4078815

[B54] Saphire-Bernstein S. Way B. M. Kim H. S. Sherman D. K. Taylor S. E. (2011). Oxytocin receptor gene (OXTR) is related to psychological resources. Proc. Natl. Acad. Sci. U.S.A. 108, 15118–15122. 10.1073/pnas.1113137108 21896752 PMC3174632

[B55] Scandurra A. Alterisio A. D'Aniello B. (2016). Behavioural effects of training on water rescue dogs in the Strange Situation Test. Appl. Anim. Behav. Sci. 174, 121–127. 10.1016/j.applanim.2015.10.007

[B56] Schöberl I. Beetz A. Solomon J. Wedl M. Gee N. Kotrschal K. (2016). Social factors influencing cortisol modulation in dogs during a strange situation procedure. J. Vet. Behav. Clin. Appl. Res. 11, 77–85. 10.1016/j.jveb.2015.09.007

[B57] Shaver P. R. Clark C. L. (1994). The psychodynamics of adult romantic attachment, in Empirical Perspectives on Object Relations Theory, eds Masling J. M. Bornstein R. F. (Washington, DC: American Psychological Association), 105–156. 10.1037/11100-004

[B58] Smeltzer M. D. Curtis J. T. Aragona B. J. Wang Z. (2006). Dopamine, oxytocin, and vasopressin receptor binding in the medial prefrontal cortex of monogamous and promiscuous voles. Neurosci. Lett. 394, 146–151. 10.1016/j.neulet.2005.10.019 16289323

[B59] Spangler G. (2011). Genetic and environmental determinants of attachment disorganization., in Disorganized Attachment and Caregiving., 110–130. Available online at: http://search.ebscohost.com/login.aspx?direct=true&db=psyh&AN=2011-16269-005&lang=ja&site=ehost-live

[B60] Thielke L. E. Udell M. A. (2015). The role of oxytocin in relationships between dogs and humans and potential applications for the treatment of separation anxiety in dogs. Biol. Rev. 92, 378–388. 10.1111/brv.12235 26548910

[B61] Topál J. Gácsi M. (2012). Lessons we should learn from our unique relationship with dogs: an ethological approach, in Crossing Boundaries, eds Birke L. Hockenhull J. (Leiden: Brill Academic Press), 163–187.

[B62] Topál J. Miklósi Á. Csányi V. Dóka A. Miklósi A. Csányi V.. (1998). Attachment behavior in dogs (*Canis familiaris*): a new application of Ainsworth's (1969) Strange Situation Test. J. Comp. Psychol. 112, 219–229. 9770312 10.1037/0735-7036.112.3.219

[B63] Turcsán B. (2017). Context and individual characteristics modulate the association between oxytocin receptor gene polymorphism and social behavior in border collies. Front. Psychol. 8:2232. 10.3389/fpsyg.2017.02232 29312078 PMC5742244

[B64] van Rooy D. Haase B. McGreevy P. D. Thomson P. C. Wade C. M. (2015). Evaluating candidate genes oprm1, drd2, avpr1a, and oxtr in golden retrievers with separation-related behaviors. J. Vet. Behav. Clin. Appl. Res. 16, 22–27. 10.1016/j.jveb.2016.03.001

[B65] Wan M. Hejjas K. Ronai Z. Elek Z. Sasvari-Szekely M. Champagne F. A.. (2013). DRD4 and TH gene polymorphisms are associated with activity, impulsivity and inattention in Siberian Husky dogs. Anim. Genet. 44, 717–727. 10.1111/age.12058 23713429

[B66] Wang J. Qin W. Liu B. Zhou Y. Wang D. Zhang Y.. (2014). Neural mechanisms of oxytocin receptor gene mediating anxiety-related temperament. Brain Struct. Funct. 219, 1543–1554. 10.1007/s00429-013-0584-9 23708061

[B67] Way B. M. Taylor S. E. (2010). Social influences on health: is serotonin a critical mediator? Psychosom. Med. 72, 107–112. 10.1097/PSY.0b013e3181ce6a7d 20145277

[B68] Windle R. J. Shanks N. Lightman S. L. Ingram C. D. (1997). Central oxytocin administration reduces stress-induced corticosterone release and anxiety behavior in rats. Endocrinology 138, 2829–2834. 10.1210/endo.138.7.5255 9202224

[B69] Yoshida M. Takayanagi Y. Inoue K. Kimura T. Young L. J. Onaka T.. (2009). Evidence that oxytocin exerts anxiolytic effects via oxytocin receptor expressed in serotonergic neurons in mice. J. Neurosci. 29, 2259–2271. 10.1523/JNEUROSCI.5593-08.2009 19228979 PMC6666325

