# Supplementary material for: Dog-Owner Attachment Is Associated With Oxytocin Receptor Gene Polymorphisms in Both Parties. A Comparative Study on Austrian and Hungarian Border Collies
Source: Front Psychol. 2018 Apr 5;9:435. doi: 10.3389/fpsyg.2018.00435 (PMC5895926; doi:10.3389/fpsyg.2018.00435)
Supplement: Supplementary file 1 [file DataSheet1.docx]

**Supplementary 1.** 44–item Big Five Inventory (OBFI_O)

Disagree strongly Disagree a little Neither agree nor disagree Agree a little Agree strongly

**1**-----------------------------**2**----------------------------------**3**-------------------------------------**4**----------------------------------**5**

| I see myself *as someone who …* |  |
| --- | --- |
| ___ 1. is talkative | ___ 23. tends to be lazy |
| ___ 2. tends to find fault with others | ___ 24. is emotionally stable, not easily upset |
| ___ 3. does a thorough job | ___ 25. is inventive |
| ___ 4. is depressed, blue | ___ 26. has an assertive personality |
| ___ 5. is original, comes up with new ideas | ___ 27. can be cold and aloof |
| ___ 6. is reserved | ___ 28. perseveres until the task is finished |
| ___ 7. is helpful and unselfish with others | ___ 29. can be moody |
| ___ 8. can be somewhat careless | ___ 30. values artistic, aesthetic experiences |
| ___ 9. is relaxed, handles stress well | ___ 31. is sometimes shy, inhibited |
| ___ 10. is curious about many different things | ___ 32. is considerate and kind to almost everyone |
| ___ 11. is full of energy | ___ 33. does things efficiently |
| ___ 12. starts quarrels with others | ___ 34. remains calm in tense situations |
| ___ 13. is a reliable worker | ___ 35. prefers work that is routine |
| ___ 14. can be tense | ___ 36. is outgoing, sociable |
| ___ 15. is ingenious, a deep thinker | ___ 37. is sometimes rude to others |
| ___ 16. generates a lot of enthusiasm | ___ 38. makes plans and follows through with them |
| ___ 17. has a forgiving nature | ___ 39. gets nervous easily |
| ___ 18. tends to be disorganized | ___ 40. likes to reflect, play with ideas |
| ___ 19. worries a lot | ___ 41. has few artistic interests |
| ___ 20. has an active imagination | ___ 42. likes to cooperate with others |
| ___ 21. tends to be quiet | ___ 43. is easily distracted |
| ___ 22. is generally trusting | ___ 44. is sophisticated in art, music, or literature |

**Big Five Inventory Scoring Key**

Extraversion: 1, 6R ^[[1]](#footnote-1)^,11, 16, 21R, 26, 31R, 36

Neuroticism: 4, 9R, 14, 19, 24R, 29, 34R, 39

Agreeableness: 2R, 7, 12R, 17, 22, 27R, 32, 37R, 42

Openness: 5, 10, 15, 20, 25, 30, 35R, 40, 41R, 44

Conscientiousness: 3, 8R, 13, 18R, 23R, 28, 33, 38, 43R

1. Note that “R” denotes reverse-scored items (1=5, 2=4, 3=3, 4=2, 5=1). [↑](#footnote-ref-1)
