# Supplementary material for: Dog-Owner Attachment Is Associated With Oxytocin Receptor Gene Polymorphisms in Both Parties. A Comparative Study on Austrian and Hungarian Border Collies
Source: Front Psychol. 2018 Apr 5;9:435. doi: 10.3389/fpsyg.2018.00435 (PMC5895926; doi:10.3389/fpsyg.2018.00435)
Supplement: Supplementary file 2 [file DataSheet2.docx]

**Supplementary 2.** 36-item Experiences in Close Relationship-Revised (ECR-R)

**Bond-related anxiety**

1. I'm afraid that I will lose my partner's love.

2. I often worry that my partner will not want to stay with me.

3. I often worry that my partner doesn't really love me.

4. I worry that romantic partners won’t care about me as much as I care about them.

5. I often wish that my partner's feelings for me were as strong as my feelings for him or her.

6. I worry a lot about my relationships.

7. When my partner is out of sight, I worry that he or she might become interested in someone else.

8. When I show my feelings for romantic partners, I'm afraid they will not feel the same about me.

9. I rarely worry about my partner leaving me.

10. My romantic partner makes me doubt myself.

11. I do not often worry about being abandoned.

12. I find that my partner(s) don't want to get as close as I would like.

13. Sometimes romantic partners change their feelings about me for no apparent reason.

14. My desire to be very close sometimes scares people away.

15. I'm afraid that once a romantic partner gets to know me, he or she won't like who I really am.

16. It makes me mad that I don't get the affection and support I need from my partner.

17. I worry that I won't measure up to other people.

18. My partner only seems to notice me when I’m angry.

**Bond-related avoidance**

19. I prefer not to show a partner how I feel deep down.

20. I feel comfortable sharing my private thoughts and feelings with my partner.

21. I find it difficult to allow myself to depend on romantic partners.

22. I am very comfortable being close to romantic partners.

23. I don't feel comfortable opening up to romantic partners.

24. I prefer not to be too close to romantic partners.

25. I get uncomfortable when a romantic partner wants to be very close.

26. I find it relatively easy to get close to my partner.

27. It's not difficult for me to get close to my partner.

28. I usually discuss my problems and concerns with my partner.

29. It helps to turn to my romantic partner in times of need.

30. I tell my partner just about everything.

31. I talk things over with my partner.

32. I am nervous when partners get too close to me.

33. I feel comfortable depending on romantic partners.

34. I find it easy to depend on romantic partners.

35. It's easy for me to be affectionate with my partner.

36. My partner really understands me and my needs.

**Scoring Information:** The first 18 items listed below comprise the attachment-related anxiety scale. Items 19 – 36 comprise the attachment-related avoidance scale. In real research, the order in which these items are presented should be randomized. Each item is rated on a 7-point scale where 1 = strongly disagree and 7 = strongly agree. To obtain a score for attachment-related *anxiety*, please average a person’s responses to items 1 – 18. However, because items 9 and 11 are “reverse keyed”. To obtain a score for attachment-related *avoidance*, please average a person’s responses to items 19 – 36. Items 20, 22, 26, 27, 28, 29, 30, 31, 33, 34, 35, and 36 will need to be reverse keyed before you compute this average.
