# Supplementary material for: Dog-Owner Attachment Is Associated With Oxytocin Receptor Gene Polymorphisms in Both Parties. A Comparative Study on Austrian and Hungarian Border Collies
Source: Front Psychol. 2018 Apr 5;9:435. doi: 10.3389/fpsyg.2018.00435 (PMC5895926; doi:10.3389/fpsyg.2018.00435)
Supplement: Supplementary file 3 [file DataSheet3.docx]

**Supplementary 3.** Eight-item Pet Avoidance and Anxiety Scales (modified ECR-R).

**Pet Avoidance Scale**

It’s easy for me to be affectionate with my pet.*

I don’t feel comfortable opening up to pets.

It helps to turn to my pet in times of need.*

I am nervous when pets get too close to me.

I find it relatively hard to get close to my pets.a

I prefer not to show a pet how I feel deep down.

I usually share my problems and concerns with my pet.b*

I feel comfortable sharing my private thoughts and feelings with my pet.*

**Pet Anxiety Scale**

I’m afraid that I will lose my pet’s love.

I am confident that my pet will want to stay with me.a*

I know that pets care about me as much as I care about them.a*

I know my pet loves me.a*

My pet makes me feel confident.a*

I find that my pets don’t want to get as close as I would like.

It makes me mad that I don’t get the affection and support I need from my pet.

My desire to be very close sometimes scares pets away.

a Original ECR-R wording was changed to balance number of secure and insecure items.

b To make this item applicable to pets, the original “discuss” was replaced with the word “share.”

* Item is reversed-scored.
